# Phytofabrication of Silver Nanoparticles Using Three Flower Extracts and Their Antibacterial Activities Against Pathogen *Ralstonia solanacearum* Strain YY06 of Bacterial Wilt

**DOI:** 10.3389/fmicb.2020.02110

**Published:** 2020-09-15

**Authors:** Hai-Jun Cheng, Hui Wang, Jing-Ze Zhang

**Affiliations:** Institute of Biotechnology, College of Agriculture and Biotechnology, Zhejiang University, Hangzhou, China

**Keywords:** *Ralstonia solanacearum*, silver nanoparticles, *Canna indica* L. flower, *Cosmos bipinnata* Cav. flower, *Lantana camara* L. flower, characterization, antibacterial activity, antibacterial mechanism

## Abstract

Bacterial wilt caused by the phytopathogen *Ralstonia solanacearum* (*R. solanacearum*) is a devastating plant disease worldwide. The use of bactericides and antibiotics for controlling bacterial wilt has shown low efficiency and posed environmental risks. This study was to phytofabricate silver nanoparticles (AgNPs) mediated by canna lily flower (*Canna indica* L.), Cosmos flower (*Cosmos bipinnata* Cav.), and Lantana flower (*Lantana camara* L.). The biosynthesized AgNPs were confirmed and characterized by UV-visible spectroscopy, Fourier transform infrared spectroscopy (FTIR), X-ray diffraction (XRD), transmission electron microscope (TEM), and scanning electron microscopy (SEM). UV-visible spectra showed absorption peak bands at 448, 440, and 428 nm of AgNPs synthesized by *C. indica* L., *C. bipinnata* Cav., and *L. camara* L. flowers, respectively. FTIR spectra confirmed that biofunctional groups of flower extract were involved in the synthesis of AgNPs as capping and stabilizing agents. The spherical AgNPs synthesized by *C. indica* L., *C. bipinnata* Cav., and *L. camara* L. flowers had average diameters of 43.1, 36.1, and 24.5 nm, respectively. The AgNPs (10.0 μg/ml) synthesized by *L. camara* L. flower had a maximum suppression zone of 18 mm against *R. solanacearum* strain YY06 compared with AgNPs synthesized by *C. indica* L. and *C. bipinnata* Cav. flowers. Bacterial growth, biofilm formation, swimming motility, efflux of nucleic acid, cell death, cell membrane damage, and reactive oxygen species (ROS) generation of *R. solanacearum* were also negatively affected by AgNPs with high concentration and small size. In summary, the biosynthesized AgNPs can be used as an efficient and environmentally friendly antibacterial agent to reasonably inhibit *R. solanacearum*.

## Introduction

Bacterial wilt caused by the phytopathogen *Ralstonia solanacearum* is a devastating plant disease worldwide, which can affect more than 200 plant species in over 50 families such as eggplants, tomatoes, olives, groundnuts, potatoes, and bananas (Hayward, 1964; Schell, 2000). The use of bactericides and antibiotics for controlling bacterial wilt has shown low efficiency due to resistant mutation of bacteria (Levy, 2001). Hence, the development of an effective and broad-spectrum antimicrobial agent is urgently required against the pathogens.

Nanotechnology is one of the evolving and most fascinating sciences, which have broad application prospects thanks to many superior properties of nanomaterials. Among the all noble metal nanoparticles, silver nanoparticles (AgNPs) occupy a prominent position due to its unique characteristics such as antibacterial, antifungal, antiviral, and anti-inflammatory properties, which can be applied to food science and anti-cancer medicine (Oves et al., 2018), especially agriculture (Oves et al., 2013).

The synthesis of AgNPs includes chemical (Sotiriou and Pratsinis, 2010; Sotiriou et al., 2011; Zhang et al., 2011; Roldán et al., 2013), physical (Tien et al., 2008; El-Nour et al., 2010; Asanithi et al., 2012), and biological (Husen and Siddiqi, 2014a; Siddiqi and Husen, 2016) routes. However, compared to biological synthesis of nanoparticle, physiochemical methods have difficulty applying on a large scale owing to the production of high temperature and harmful chemicals (Ahmed et al., 2016).

Biological stuff such as plants, bacteria, and fungi were used for green synthesis of AgNPs (Castro-Longoria et al., 2011; Husen and Siddiqi, 2014b), especially plants are more advantageous because of less contamination threat and easy availability. Recent studies have reported that AgNPs can be synthesized by using various plants parts such as leaves extract of *Nigella sativa* (Amooaghaie et al., 2015), roots extract of red ginseng (Singh et al., 2015b), seeds extract of *Pistacia atlantica* (Sadeghi et al., 2015), flowers extract of *Nyctanthes arbortristis* (Gogoi et al., 2015), fruits extract of orange and pineapple (Hyllested et al., 2015), etc. *Canna indica* L. have been reported to use as diaphoretic and diuretic in fevers and dropsy, as a demulcent to stimulate menstruation, treat suppuration, and rheumatism (Duke and Ayensu, 1984). *Cosmos bipinnata* Cav. is used traditionally as a hepatoprotective agent and for the management of leukemia, headache, jaundice, splenomegaly, stomach aches, flatulence, and intermittent malarial fever (Jo et al., 2012; Olajuyigbe and Ashafa, 2014). *Lantana camara* L. is used in medicine against influenza, measles, stomachache, chicken pox, fever, rheumatisms, asthma, and hypertension (Kumar et al., 2015). In addition, the major functional molecules of three plants extract include alkaloids, carbohydrates, proteins, flavonoids, terpenoids, steroids, saponins, and tannins (Ghisalberti, 2000; Wang et al., 2007; Saleem et al., 2019) as capping and stabilizing agents play a significant role for the synthesis of nanoparticles (Duan et al., 2015).

At the same time, we have limited knowledge about antibacterial mechanisms of nano-silver against *R. solanacearum* and need to continue to investigate it. Therefore, the work of this research was mainly to green synthesize AgNPs mediated by Canna lily flower (*C. indica* L.), Cosmos flower (*C. bipinnata* Cav.), and Lantana flower (*L. camara* L.) and demonstrate the antibacterial activities and mechanisms against the pathogen *R. solanacearum* of bacterial wilt. To the best of our knowledge, this is the first report to synthesize AgNPs using flower extract of *C. indica* L. and *C. bipinnata* Cav.

## Materials and Methods

### Materials

Three plants namely Canna lily flower (*C. indica* L.), Cosmos flower (*C. bipinnata* Cav.), and Lantana flower (*L. camara* L.), were collected from Zijingang campus, Zhejiang University, Hangzhou, China. The analytical grade silver nitrate (AgNO_3_) was purchased from Sinopharm Chemical Reagent Co., Ltd. company (Shanghai, China). The bacterial wilt pathogen *R. solanacearum* strain YY06 (a highly aggressive strain from eggplant) was provided from the Institute of Biotechnology, Zhejiang University, Hangzhou, China. The bacteria were routinely cultured in nutrient agar (NA) medium composed of beef extract 3 g, NaCl 5 g, peptone 10 g, ddH_2_O 1,000 ml, without/with agar 15 g, pH 7.2–7.4 at 37°C.

### Preparation of Flower Extracts

Flower extracts of *C. indica* L., *C. bipinnata* Cav., and *L. camara* L. were prepared following the procedure of Moteriya and Chanda (2017) with small adjustments. Briefly, the flowers were carefully rinsed with ddH_2_O and then crushed into small pieces. Two gram air-dried flowers were stirred with a blender in 200 ml ddH_2_O to acquire 1% (w/v) flower broth. The resulting extracts were filtered with a muslin cloth, centrifuged for 10 min at 14,000 rpm, and retained at 4°C.

### Synthesis of AgNPs Using Different Flower Extracts

According to Gogoi et al. (2015) with minor modifications, 20 ml aqueous flower extract was mixed with 100 ml AgNO_3_ solution (4 mM) in an Erlenmeyer flask, which was then kept under the dark condition at rotating 180 rpm at 55°C. AgNO_3_ solution was mixed with ddH_2_O instead of flower extracts, which was used a control. When the color of the solution became dark brown, it indicated that the silver ions had been reduced to AgNPs. The produced AgNPs solution was centrifuged (JEOL, JEM-200EX; Tokyo, Japan) at 14,000 rpm for 10 min and removed the supernatant. The collected pellets were carefully washed with ddH_2_O and freeze-dried according to Alpha1-2 LDplus (GmbH, Germany) instruction and then saved at −4°C for further use.

### Confirmation and Characterization of Synthesized AgNPs

The collected pellets were measured under the wavelength range of 300–700 nm at 1 nm resolution in a spectrophotometer (Shimadzu Corporation, Kyoto, Japan). The infrared spectra of the dried AgNPs was recorded at room temperature by a Fourier transform infrared spectroscopy (FTIR) machine (Vector 22, Bruker, Germany) scanned at a resolution of 4 cm^−1^ and range between 450 and 4,000 cm^−1^. The X-ray diffraction (XRD) value of nanoparticles was obtained by using a diffractometer (Siemens D5000, Germany) at 2Ɵ range of 20–90°. Scanning electron microscopy (SEM; SU8010, Hitachi, Japan) and transmission electron microscope (TEM; JEM-1230, JEOL, Akishima, Japan) were used to document the size and shape of AgNPs according to the operating instruction.

### Inhibition Zone Assay

The effect of AgNPs on the *R. solanacearum* strain YY06 was investigated by plate assay as depicted by Singh et al. (2015a) with few amendments. Four hundred microliter bacterial suspension (1 × 10^8^ CFU/ml) cultured overnight in NA broth at 37°C was mixed with 10 ml unsolidified NA in a Petri dish plate. Forty microliter AgNPs suspension (2.5, 5.0, and 10.0 μg/ml) was, respectively, dropped in holes of the air-dried NA plate and incubated at 37°C for 24 h. The same quantity of the filter-sterilized flower extract was used as control. The antagonistic activity was estimated by averaging the diameter of the cleared zones. The experiment was performed three times with three replications.

### Minimum Inhibitory Concentration of AgNPs

A broth dilution procedure was used to assess the minimum inhibitory concentration (MIC) of AgNPs in a 96-well microplate (Corning-Costar Corp, Corning, NY, United States). According to Mohanta et al. (2017) with slight modifications, 100 μl overnight bacterial broth (approximately 1 × 10^8^ CFU/ml) was mixed with 100 μl AgNPs suspension of 2-fold serial dilutions (2.5, 5.0, and 10.0 μg/ml) followed by culturing at 37°C for 24 h, bacterial broth culture without AgNPs was used as control. A microplate spectrophotometer (Thermo Fisher Scientific Inc., Waltham, MA) was used to measure the optical density of the mixed solution at 600 nm. There were three replicates for each treatment and three repeats for each experiment.

### Biofilm Inhibition Assay

The effect of AgNPs on bacterial biofilm formation was determined on the 96-well microplate (Corning-Costar Corp., Corning, NY). According to Ibrahim et al. (2019) with slight adjustments, *R. solanacearum* strain YY06 suspension adjusted to OD600 = 0.3 (100 μl) was transferred into each well of the 96-well microplate with 100 μl of suspension without or with AgNPs. The microplate was placed without shaking at 37°C for 3 days. After bacterial adhesion, the suspension was discarded followed by a gentle wash with ddH_2_O three times to remove the free cells. As soon as air was dry, an aqueous solution of 1% crystal violet (100 μl) was transferred into each well and the 96-well microplate placed for half an hour to stain the attached bacteria. The dye was discarded and wells of the microplate were subsequently washed gently with ddH_2_O. A 33% acetic acid (100 μl) was then added to dissolve the dye, and the intensity of the reaction mixture was measured using a spectrophotometer at 570 nm.

### Swimming Motility Assay

The impact of AgNPs on swimming motility of *R. solanacearum* strain YY06 was tested on semi-solid NA [0.4% (w/v)]. AgNPs were mixed into the soft agar medium to obtain a final concentration of 2.5, 5.0, and 10.0 μg/ml. Three microliter *R. solanacearum* strain YY06 suspension at a concentration of 10^8^ CFU/ml was dropped in the center of the semi-solid NA plates containing AgNPs or no AgNPs, which were incubated at 37°C for 48 h. The migration diameter of *R. solanacearum* strain YY06 was measured to assess the swimming ability due to the exposure to AgNPs. There were three replicates for each treatment and three repeats for each experiment.

### Efflux of the Cytoplasmic Materials

Upon damage to the cell membrane, substances (DNA and RNA) were released out of the cell, which was documented the optical density of the solution at 260 nm. As described by Cai et al. (2018) with slight modifications, the 1,000 μl overnight bacterial suspension with AgNPs (2.5, 5.0, and 10.0 μg/ml) was cultured at 37°C for 12 h. The samples were then centrifuged at 5,000 *g* for 4 min to remove the bacteria and AgNPs. OD value of the solution was noted at 260 nm ultraviolet light using a spectrophotometer.

### Live/Dead Assay

BacLight bacterial viability kit (Invitrogen), a mixture of red propidium iodide fluorescent nucleic acid dye and green SYTO 9 fluorescent nucleic acid dye, was applied to detect live/dead bacterial cells. One thousand microliter overnight bacterial suspension (1 × 10^8^ CFU/ml) was mixed with 1 ml solution of AgNPs to acquire a concentration of 10.0 μg/ml and was subsequently incubated in a shaker at 180 rpm, 37°C. Fluorescence emission of bacteria was recorded by using a laser scanning confocal microscope (LSM780) as mentioned before (Cui et al., 2014).

### Flow Cytometry Observation

Propidium iodide (PI) is a type of fluorochrome that can just penetrate the compromised cell and embed in double-stranded nucleic acid. Therefore PI can be applied to assess the damage of the cell membrane upon exposure to nanoparticles (Kumar et al., 2011). The *R. solanacearum* strain YY06 suspension treated with AgNPs (10.0 μg/ml) was incubated in a rotary shaker with 180 rpm at 37°C for 36 h and subsequently dyed with propidium iodide (PI) in the dark for half an hour. The staining cells can be monitored using flow cytometry (BD FACSVerse).

### Electron Microscopy Analysis

The cellular adsorption assay was conducted using the method described in the preceding studies (Cai et al., 2018; Abdallah et al., 2019). One thousand microliter *R. solanacearum* strain YY06 suspension (1 × 10^8^ CFU/ml) was mixed with 10 μg/ml AgNPs mediated by *L. camara* L. The bacterial suspensions treated with or without the AgNPs were put in a shaker with 180 rpm at 37°C for 12 h. After centrifugated at 5,000 *g* for 4 min, the bacterial cells were washed three times with 0.1 mol/l phosphate buffered saline (PBS) followed by fixing with 2.5% (v/v) glutaraldehyde in 0.1 M PBS. The samples were then post-fixed with 1% (w/v) osmium tetroxide in 0.1 M PBS for 1 h followed by washing three times with 0.1 M PBS buffer. The samples were dehydrated with a range of ethanol solutions (50, 70, 80, 90, 95, and 100%). TEM (JEM-1230, JEOL, Akishima, Japan) and SEM (SU8010, Hitachi, Japan) were used to document the changes of bacteria according to the operating instruction. The energy dispersive X-ray spectroscopy (EDS; Hitachi, Japan) was used to detect the elements of the cell surface.

### Determination of the Reactive Oxygen Species

DCFH-DA is a non-polar dye that can be converted to polar derivative DCFH by cellular esterase; non-fluorescent DCFH reacts further with ROS to form the DCF which can fluoresce at a wavelength of 488 nm (Applerot et al., 2012). To detect the intracellular ROS, 1,000 μl *R. solanacearum* strain YY06 suspension (1 × 10^8^ CFU/ml) was mixed with 10 μg/ml AgNPs mediated by *L. camara* L. and cultured in a rotary shaker with 180 rpm at 37°C for 12 h. The bacterial suspension not treated with AgNPs was used as the experimental control. Subsequently, the treated samples were gently rinsed three times with sterilized water and inoculated with 10 mM DCFH-DA at 37°C for 30 min in the dark. The fluorescence was detected by using the laser scanning confocal microscope (LSM780) as described previously (Prasad et al., 2019). The experiment was repeated three times.

### Statistical Analysis

The ANOVA test was analyzed by the software SPSS21 (America), mean values of treatments were done using the least significant difference (LSD) method at *p* < 0.05.

## Results and Discussion

### Biosynthesis and Characterization of AgNPs

**Figure 1:**
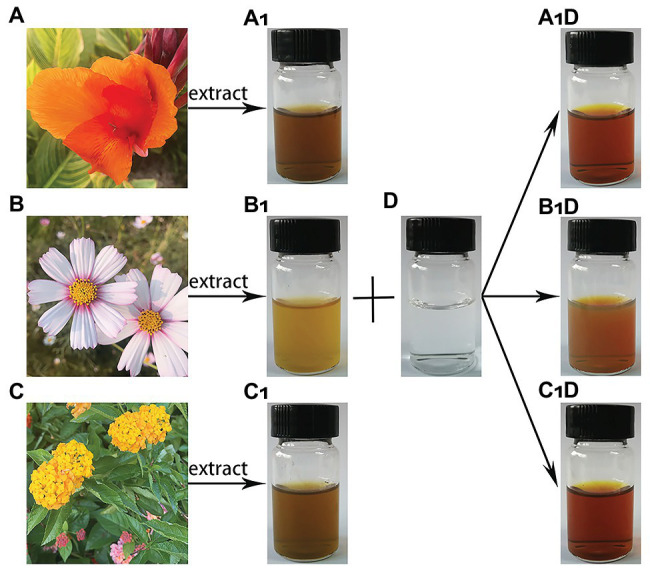
Schematic presentation of silver nanoparticles (AgNPs) synthesis. **(A)** *Canna indica* L. flower; **(B)** *Cosmos bipinnata* Cav. flower; **(C)** *Lantana camara* L. flower; **(A_1_)** filtered *C. indica* L. flower aqueous extract; **(B_1_)** filtered *C. bipinnata* Cav. flower aqueous extract; **(C_1_)** filtered *L. camara* L. flower aqueous extract; **(D)** silver nitrate solution; **(A_1_D)** solution AgNPs synthesized by *C. indica* L. flower; **(B_1_D)** solution AgNPs synthesized by *C. bipinnata* Cav. flower; and **(C_1_D)** solution AgNPs synthesized by *L. camara* L. flower.

The synthesis of AgNPs through the aqueous flower extracts of *C. indica* L., *C. bipinnata* Cav., and *L. camara* L. was as a result of the reduction of silver ions (Ag^+^) to Ag^0^ in AgNO_3_ (4 mM) solution at the optimum temperature (Figure 1). The change of the solution color to dark brown indicated that the silver ions have been reduced to AgNPs (Nayak et al., 2016). The UV-visible spectra of nanoparticles mediated by *C. indica* L., *C. bipinnata* Cav., and *L. camara* L. flowers showed absorption peak bands at 448, 440, and 428 nm, respectively (Supplementary Figure S1). Similar results of wavelength were obtained for *Iresine herbstii* leaf and *Erigeron annuus* flower aqueous extracts mediated synthesis of AgNPs (Dipankar and Murugan, 2012; Velmurugan et al., 2014).

The FTIR spectra of AgNPs derived from *C. indica* L., *C. bipinnata* Cav., and *L. camara* L. flowers are illustrated in Supplementary Figure S2. The main band intensity was as follows: 3,421, 3,419, and 3,416 cm^−1^ corresponded to N−H and O−H stretching vibrations of amine groups. Bands at 2,919, 2,918, and 2,920 cm^−1^ appeared due to stretching vibration of C−H, and the bands at 1,598, 1,636, and 1,649 cm^−1^ were assigned to C〓O amide groups. The bands at 1,309, 1,384, and 1,384 cm^−1^ represented COO− anions and the spectra bands at 1,070, 1,072, and 1,048 cm^−1^ appeared due to the C−N stretching vibration. The infrared spectra of AgNPs revealed that the flower extracts performed a dual role in the solution mixture, i.e., as a reducing agent of Ag^+^ and stabilizing agent of Ag with the biological functional groups. Carboxylic acids, ketones, aldehydes, and amine linked to the silver ions reduction, which stabilize nanoparticles due to the oxidation of hydroxyl radical (Aziz et al., 2019).

The XRD image of three synthesized AgNPs was shown in Supplementary Figure S3. The synthesized AgNPs from *C. indica* L. flower had diffraction peaks at 38.137, 44.274, 64.504, and 77.370, while the AgNPs derived from *C. bipinnata* Cav. flower showed diffractions peaks at 38.076, 44.171, 64.442, and 77.472. On the other hand, the AgNPs synthesized by *L. camara* L. flower emitted diffraction peaks at 38.158, 44.274, 64.504, and 77.431, corresponding to the silver crystal planes (111), (200), (220), and (311), which revealed that AgNPs had varied face-centered cubic (fcc) planes in agreement with earlier reports (Ma et al., 2012; Velmurugan et al., 2015).

**Figure 2:**
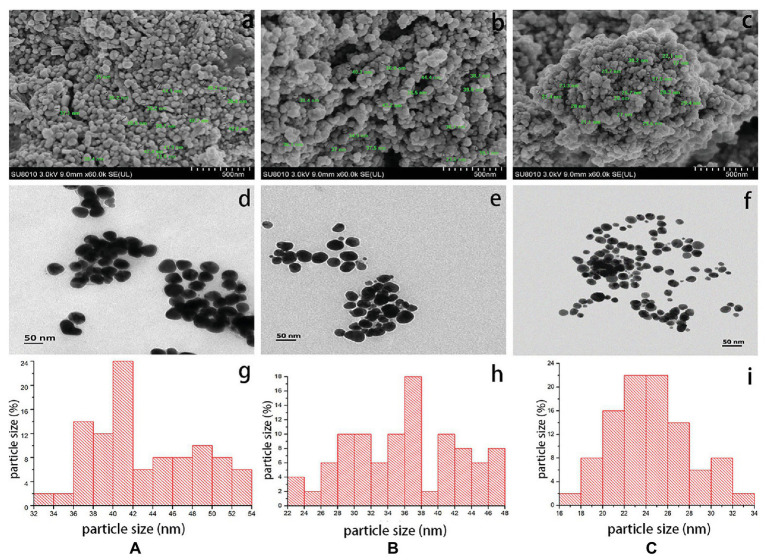
Scanning electron micrographs of AgNPs synthesized by **(a)** *C. indica* L. flower; **(b)** *C. bipinnata* Cav. flower; and **(c)** *L. camara* L. flower; scale bar = 500 nm. Transmission electron micrographs of AgNPs synthesized by **(d)** *C. indica* L. flower; **(e)** *C. bipinnata* Cav. flower; and **(f)** *L. camara* L. flower; scale bar = 50 nm. The size distribution of particles in transmission electron micrographs of AgNPs synthesized by **(g)** *C. indica* L. flower; **(h)** *C. bipinnata* Cav. flower; and **(i)** *L. camara* L. flower.

Herein, electron micrographs of AgNPs synthesized by the flowers were shown in Figure 2, the TEM micrographs indicated that AgNPs were mono-dispersed in irregularly spherical particle-like shapes, which conformed to the pictures of the SEM. The TEM images showed that AgNPs had average diameters of 43.1, 36.1, and 24.5 nm synthesized by *C. indica* L., *C.bipinnata* Cav., and *L. camara* L. flowers, respectively. The SEM images showed that the diameters ranged from 28.7 to 45.5 nm for AgNPs synthesized by *C. indica* L. flower, 33.5 to 44.4 nm for AgNPs synthesized by *C. bipinnata* Cav. flower, and 21.1 to 30.2 nm for AgNPs synthesized by *L. camara* L. flower. In the study, we observed the AgNPs clusters that may cause the difference of particle size, which were reported by previous studies (Rasheed et al., 2017).

### Antibacterial Activities

**Figure 3:**
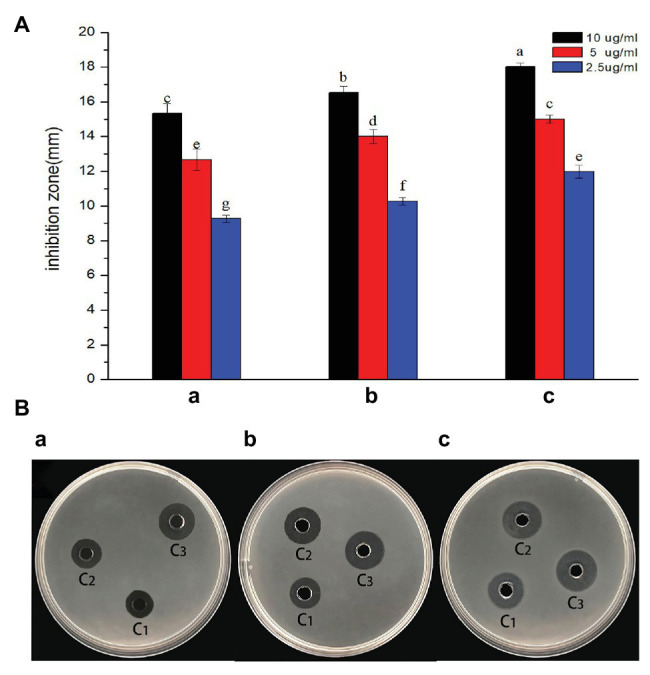
*In vitro* inhibitory effect of AgNPs synthesized by flower extract against *R. solanacearum* strain YY06. **(a)** Diameter of bacterial growth inhibition (mm) caused by AgNPs; **(b)** bacterial growth inhibition zone caused by AgNPs, which were synthesized by (A) *C. indica* L. flower; (B) *C. bipinnata* Cav. flower; and (C) *L. camara* L. flower; C1 = 2.5 μg/ml, C2 = 5 μg/ml, and C3 = 10 μg/ml. Vertical bars represent standard errors of the means (*n* = 3). Bars followed by the same letter(s) are not significantly different in least significant difference (LSD) test (*p* ≤ 0.05).

The antagonistic activity of AgNPs against *R. solanacearum* strain YY06 was determined by measuring the bacterial growth inhibition zone formation in solid agar media. The result showed that the diameter of the bacteriostatic zone enlarged with the increase in concentration of AgNPs having its maximum antibacterial effect at 10.0 μg/ml. The AgNPs synthesized by *L. camara* L. flower had the highest bacteriostatic zone of 18 mm against *R. solanacearum* strain YY06 compared with AgNPs synthesized by *C. indica* L. and *C. bipinnata* Cav. flowers (Figure 3), while the control flower aqueous extract had no inhibition zone (data not shown). We also tested the inhibitory activity of AgNPs against *Xanthomonas oryzae* pv. oryzae (Xoo) strain GZ 0003, which had a good bacteriostatic effect (Supplementary Figure S4). Silver nanoparticle as a bacteriostatic agent was reported previously on *Escherichia coli*, *Pseudomonas aeruginosa*, *Bacillus subtilis*, and *Staphylococcus aureus* (Ahmed et al., 2016).

**Figure 4:**
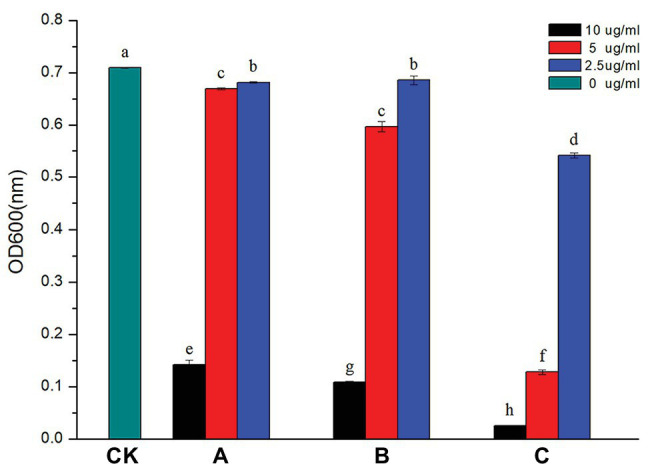
Effect of AgNPs on the growth of *R. solanacearum* strain YY06. AgNPs mediated by (A) *C. indica* L. flower; (B) *C. bipinnata* Cav. flower; and (C) *L. camara* L. flower. Vertical bars represent standard errors of the means (*n* = 3). Bars followed by the same letter(s) are not significantly different in LSD test (*p* ≤ 0.05).

The MIC of AgNPs on *R. solanacearum* strain YY06 was tested on a 96-well microplate. As shown in Figure 4, the bacterial number reduced by 3.93, 3.33, and 23.64% at 2.5 μg/ml, and 5.62, 15.88, and 81.89% at 5.0 μg/ml owing to exposure of AgNPs synthesized by *C. indica* L., *C. bipinnata* Cav., and *L. camara* L., respectively. On the other hand, AgNPs synthesized by *L. camara* L. at 10.0 μg/ml had the highest reduction of OD600 by 96.40% compared to the other synthesized AgNPs. The nano-size particles with high surface area, strong adsorption, and positive charge enable them to effectively inhibit bacterial growth (Wang et al., 2017). Furthermore, the result revealed that the higher concentration and smaller size of nanoparticles, the greater impact on bacterial growth as described previously (Kim et al., 2007).

**Figure 5:**
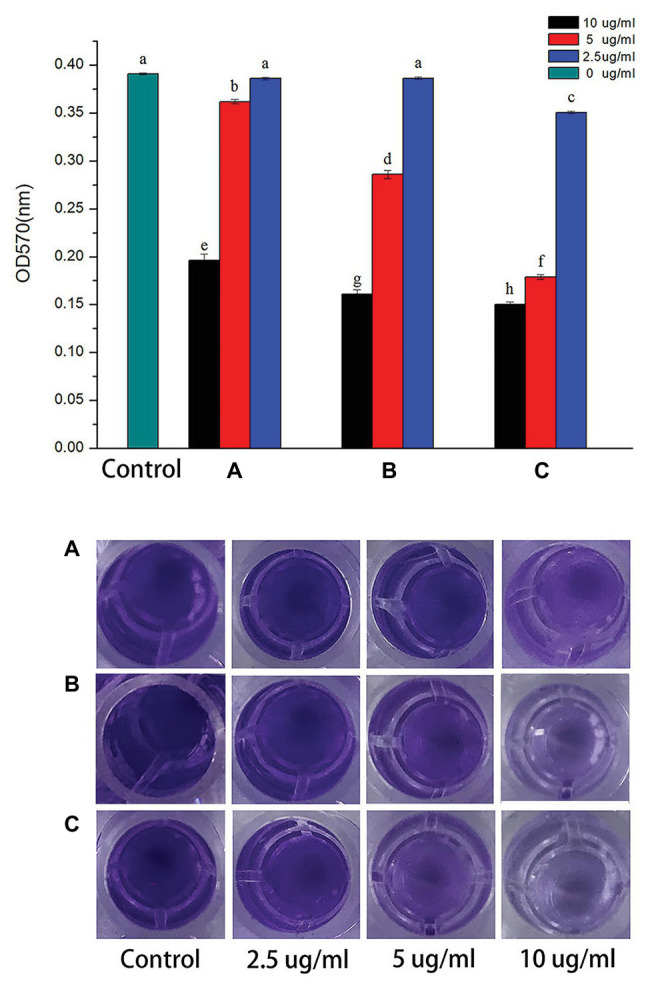
Effect of AgNPs on biofilm formation of *R. solanacearum* strain YY06. AgNPs synthesized by **(A)** *C. indica* L. flower; **(B)** *C. bipinnata* Cav. flower; and **(C)** *L. camara* L. flower. Vertical bars represent standard errors of the means (*n* = 3). Bars followed by the same letter(s) are not significantly different in LSD test (*p* ≤ 0.05).

The biofilm formation played an important role in the virulence of plant pathogen by increasing the survivability of bacteria in harsh conditions, resisting to bacteriostatic substances derived from plants and motivating colonization in the host plants (Dang and Lovell, 2016). In this study, it was evaluated whether the synthesized AgNPs (2.5, 5.0, and 10.0 μg/ml) could inhibit the biofilm formation of *R. solanacearum* strain YY06 by staining with crystal violet in 96-well plates. As shown in Figure 5, with the increase of AgNPs concentration, the color of solution in wells gradually lightened, which indicated that the biomass of biofilm decreased. The AgNPs synthesized by *C. indica* L., *Cosmos bipinnata* Cav., and *L. camara* L. flowers decreased the biofilm formation of *R. solanacearum* strain YY06 by 49.85, 58.78, and 61.55% at 10.0 μg/ml, compared to the control OD570 value 0.39 of *R. solanacearum* strain YY06 without AgNPs.

**Figure 6:**
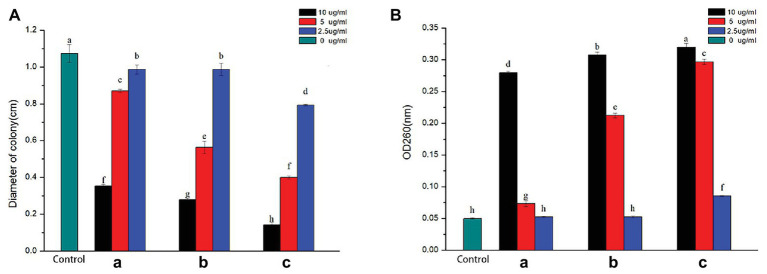
**(a)** Effect of AgNPs on the swimming motility of *R. solanacearum* strain YY06. **(b)** Effect of AgNPs on the efflux of cytoplasmic materials of *R. solanacearum* strain YY06. AgNPs synthesized by (A) *C. indica* L. flower; (B) *C. bipinnata* Cav. flower; and (C) *L. camara* L. flower. Vertical bars represent standard errors of the means (*n* = 3). Bars followed by the same letter(s) are not significantly different in LSD test (*p* ≤ 0.05).

Swimming motility of bacteria is directly involved in its growth and pathogenesis, crucial for surface adherence, structural disassembly, and discharge from the matrix of biofilm (Klausen et al., 2003). To assess the effect of AgNPs on bacterial movement, the diameter of the vicinity covered by *R. solanacearum* strain YY06 was measured on soft NA medium supplied with AgNPs (2.5, 5.0, and 10.0 μg/ml) after 24 h of incubation. The result showed that compared with the control, 10.0 μg/ml AgNPs synthesized by *C. indica* L., *C. bipinnata* Cav., and *L. camara* L. led to a significant reduction in the halo diameter by 67.08, 73.91, and 86.65%, respectively (Figure 6A). Although the AgNPs at 2.5 and 5.0 μg/ml inhibited the bacterial movement somewhat but statistically less than the swimming inhibition for AgNPs at 10 μg/ml.

When the cell membranes ruptured, DNA and RNA were released out of the cell, which could be documented at an optical density of 260 nm (Chen et al., 2013). The value of nucleic acid efflux of *R. solanacearum* strain YY06 after inoculated AgNPs for 12 h at 30°C was shown in Figure 6B. Compared to the OD260 value of control treatment of 0.05, AgNPs mediated by *C. indica* L., *C. bipinnata* Cav., and *L. camara* L. at 10.0 μg/ml had an OD260 value of 0.28, 0.31, and 0.32 nm, respectively, which indicated that the cell membrane was seriously damaged.

### Bacterial Death Measurement

**Figure 7:**
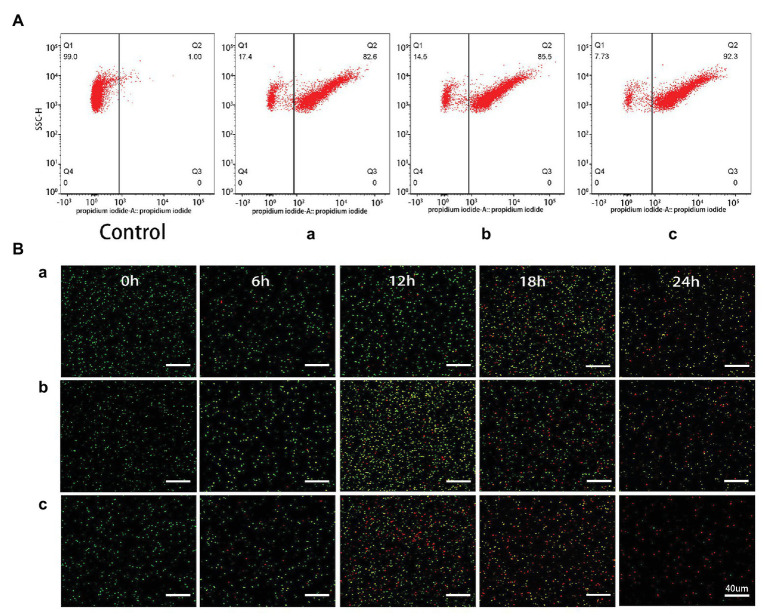
**(a)** Flow cytometry images of *R. solanacearum* strain YY06 cells after 36 h incubation without (CK) and with AgNPs synthesized by (A) *C. indica* L. flower; (B) *C. bipinnata* Cav. flower; and (C) *L. camara* L. flower. **(b)** Confocal fluorescence microscopic images of *R. solanacearum* strain YY06 cells at different times (0, 6, 12, 18, and 24 h) treated with AgNPs synthesized by (A) *C. indica* L. flower; (B) *C. bipinnata* Cav. flower; and (C) *L. camara* L. flower. Cells with green fluorescence represent live bacteria, whereas red cells are representative of dead bacteria, scale bar = 40 μm.

After a short period of staining with the live/dead viability kit, dead or dying cells with damaged membranes could be stained red, while alive cells with intact membranes could be stained green. Figure 7B showed the fluorescence pictures of bacterial cells when exposed to AgNPs (10.0 μg/ml) for 0, 6, 12, 18, and 24 h. At the beginning of adding AgNPs, almost all of the cells remained alive which can be deduced from the green fluorescence. As the incubation time increased, the images showed increasing red fluorescence. The AgNPs (10.0 μg/ml) synthesized *L. camara* L. resulted in the largest decrease in the number of living bacteria and total bacteria at 24 h compared with AgNPs synthesized by *C. indica* L. and *C. bipinnata* Cav.

Flow cytometry test was conducted for the rapid detection of the living and dead cells (Berney et al., 2007). Here, the bacterial death caused by AgNPs was examined based on the flow cytometric analysis in combination with PI. As shown in Figure 7A, the death rate of *R. solanacearum* strain YY06 increased by 82.5, 85.5, and 92.3% in comparison with the control (1%) after treated for 36 h with 10 μg/ml AgNPs synthesized by *C. indica* L., *C. bipinnata* Cav., and *L. camara* L., respectively. The result showed that the AgNPs improved the permeability of the *R. solanacearum* strain YY06 cell membrane resulting in injury and ultimately cell death, which was also observed in the live/dead cell staining test.

### The Mechanism of Silver Nanoparticles Against *R. solanacearum*

**Figure 8:**
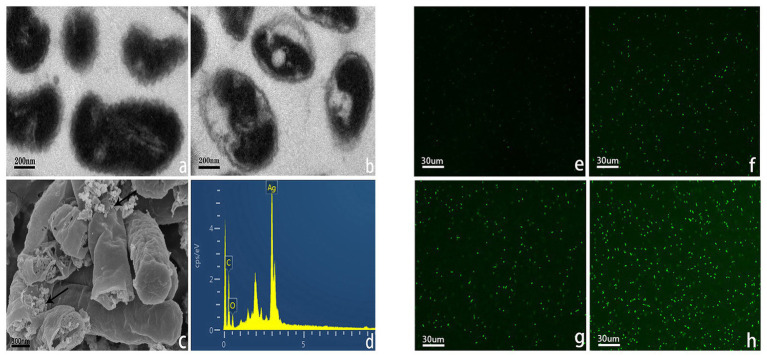
Transmission electron microscope (TEM) images of *R. solanacearum* strain YY06 treated without **(a)** and **(b)** 10 μg/ml of AgNPs synthesized by *L. camara* L. flower, scale bar = 200 nm. Scanning electron microscopy (SEM) images of *R. solanacearum* strain YY06 **(c)** treated with 10 μg/ml AgNPs synthesized by *L. camara* L. flower, arrows point to AgNPs; with **(d)** energy dispersive X-ray spectroscopic (EDS) analysis of particles on the surface of the cells, scale bar = 300 nm. Formation of reactive oxygen species (ROS) in *R. solanacearum* strain YY06 cells after a 12 h incubation period without **(e)** and with 10.0 μg/ml AgNPs synthesized by **(f)** *C. indica* L. flower; **(g)** *C. bipinnata* Cav. flower; and **(h)** *L. camara* L. flower, scale bar = 30 μm.

TEM can be applied to explore the ultrastructural changes of cell, and SEM is usually used to observe surface of the external cell (Sun et al., 2017). In this study, the images of SEM and TEM revealed the changes of *R. solanacearum* strain YY06 cells after incubating in the nutrient broth with 10 μg/ml AgNPs mediated by *L. camara* L. The integrity of cells grown without AgNPs remained intact with dense cytoplasm filled in the bacteria (Figure 8A). In contrast, the bacterial cells exposed to the AgNPs were severely disrupted and twisted while cytoplasm was shrunk (Figure 8B). In addition, we hypothesized that AgNPs powders adhered to the walls of the bacterial cells and obtained the expected results. According to EDS analysis (Figures 8C,D), there was a peak of the Ag element on the surface of the bacteria exposed to AgNPs, whereas the control cell remained without any Ag element (data not shown).

As far as we know, *R. solanacearum* is a kind of negatively charged Gram-negative bacteria that has an electrostatic attraction to positively charged nanoparticles (Leung et al., 2014). Furthermore, compared to chemically synthesized nanoparticles, green nanoparticles can adhere to the membrane of bacteria more fastly and firmly (Sudhasree et al., 2014; Happy et al., 2018).

ROS such as superoxide (O_2_^−^), hydroxyl (OH), peroxy (RCOO), and hydrogen peroxide (H_2_O_2_; Gülçin et al., 2011) can destroy cell structures including lipid peroxidation, DNA oxidative damage, protein denaturation, and enzyme inactivation (Birben et al., 2012), leading to bacterial decomposition and death (Mendis et al., 2005; Madl et al., 2014). The fluorescence intensity of *R. solanacearum* strain YY06 increased significantly after treated with 10 μg/ml AgNPs for 12 h in comparison with control (without AgNPs) in Figures 8E–H. Cells exposed to AgNPs synthesized by *L. camara* L. flower had higher intensity of fluorescence significantly more than AgNPs synthesized by *C. indica* L. and *C. bipinnata* Cav. flowers.

Most bacteria are divided into Gram-positive and Gram-negative on the basis of the cell wall structure. Gram-positive bacteria contain a thick peptidoglycan layer of the cell wall, while the peptidoglycan layer of Gram-negative bacteria wall is thin. Many studies have found that Gram-positive bacteria are more resistant to nanoparticle than Gram-negative bacteria (Velmurugan et al., 2017). The thick peptidoglycan layer of Gram-positive bacteria may act as a protective function when exposing to nanoparticles. Another reason is that the negatively charged Gram-negative bacteria have a higher affinity for the positive ions released by nanoparticles, resulting in accumulation and absorption of ions, which cause intracellular damage (Feng et al., 2000; Kim et al., 2012; Mukha et al., 2013; Cavassin et al., 2015; Dorobantu et al., 2015).

Secondary metabolites such as alkaloids, carbohydrates, proteins, flavonoids, terpenoids, steroids, saponins, and tannins from plants acted as capping and stabilizing agents responsible for the synthesis of AgNPs and control of the particle size (Duan et al., 2015). However, due to complex structure and variety of functional molecules, what reason causes the different sizes of AgNPs mediated by three plants requires further research. AgNPs as an antibacterial agent can prevent the growth of plant pathogens but at the same time may kill beneficial microorganisms. In recent years, the impact of AgNPs on environmental safety and human health have led to public concerns (Lansdown, 2006). Current reports on AgNPs are mainly *in vitro* studies, and *in vivo* studies are still not sufficiently documented due to lack of safe environmental protocol. In our research, AgNPs showed low cytotoxicity to mammalian cells (Supplementary Figure S5). In previous studies, Mirzajani et al. (2013) revealed that AgNPs could not penetrate the root cells of rice when the concentration of AgNP was less than 30 μg/ml, while AgNPs above 30 μg/ml can damage the cell membrane and produce side effect that had an impact on root growth. Therefore, more in-depth research will be needed in the interaction of the plant-microbe-nano system to grasp clear agricultural consequences of AgNPs.

## Conclusion

In this work, AgNPs were successfully phytofabricated using extracts of Canna lily flower (*C. indica* L.), Cosmos flower (*C. bipinnata* Cav.), and Lantana flower (*L. camara* L.). The biosynthesized AgNPs were confirmed and characterized by UV-visible spectroscopy, FTIR, XRD, and electron microscopy (TEM and SEM). The results of antagonistic experiments showed that AgNPs synthesized by *L. camara* L. flower had the highest impact on the bacterial growth, biofilm formation, swimming motility, efflux of nucleic acid, cell death, cell membrane damage, and ROS generation of *R. solanacearum*, which were ascribed to the particle size of 24.5 nm smaller than other AgNPs. Therefore, our study concluded that the particle size of AgNPs played a very important role in their antibacterial activities. To sum up, the biosynthesized AgNPs can be used as an efficient and environmentally friendly antibacterial agent to reasonably inhibit *R. solanacearum*.

## Data Availability Statement

The raw data supporting the conclusions of this article will be made available by the authors, without undue reservation.

## Author Contributions

H-JC and J-ZZ designed the experiment. H-JC and HW carried out the experiment. H-JC wrote the manuscript. J-ZZ improved the manuscript. All authors contributed to the article and approved the submitted version.

### Conflict of Interest

The authors declare that the research was conducted in the absence of any commercial or financial relationships that could be construed as a potential conflict of interest.
