# Supplementary figures and images for: Phytofabrication of Silver Nanoparticles Using Three Flower Extracts and Their Antibacterial Activities Against Pathogen Ralstonia solanacearum Strain YY06 of Bacterial Wilt
Source: Front Microbiol. 2020 Sep 15;11:2110. doi: 10.3389/fmicb.2020.02110 (PMC7522305; doi:10.3389/fmicb.2020.02110)

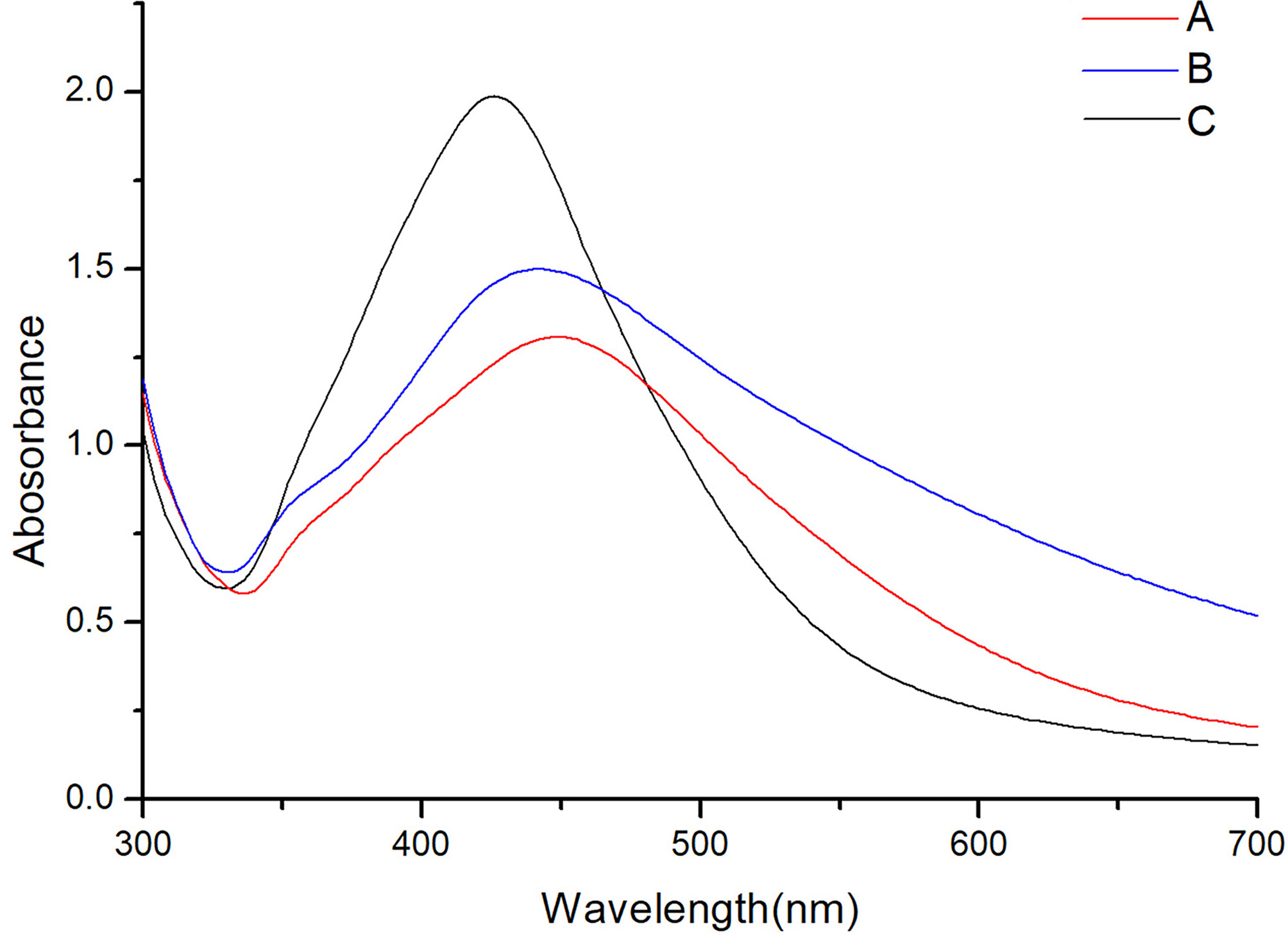

Supplement: Supplementary file 1 [file Data_Sheet_1.zip › supplymentary materials/supplementary Figure/Figure S1.jpg]

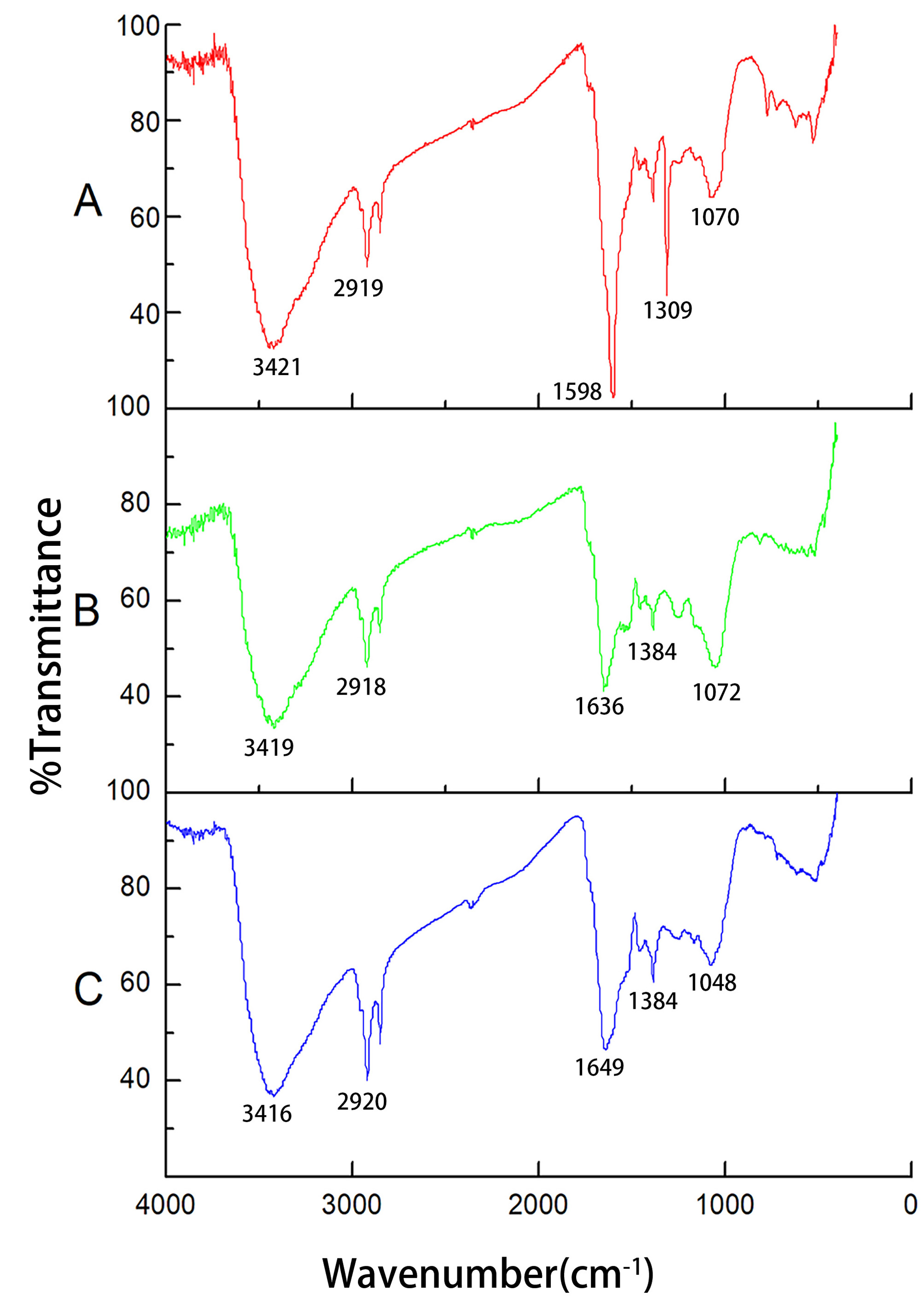

Supplement: Supplementary file 1 [file Data_Sheet_1.zip › supplymentary materials/supplementary Figure/Figure S2.jpg]

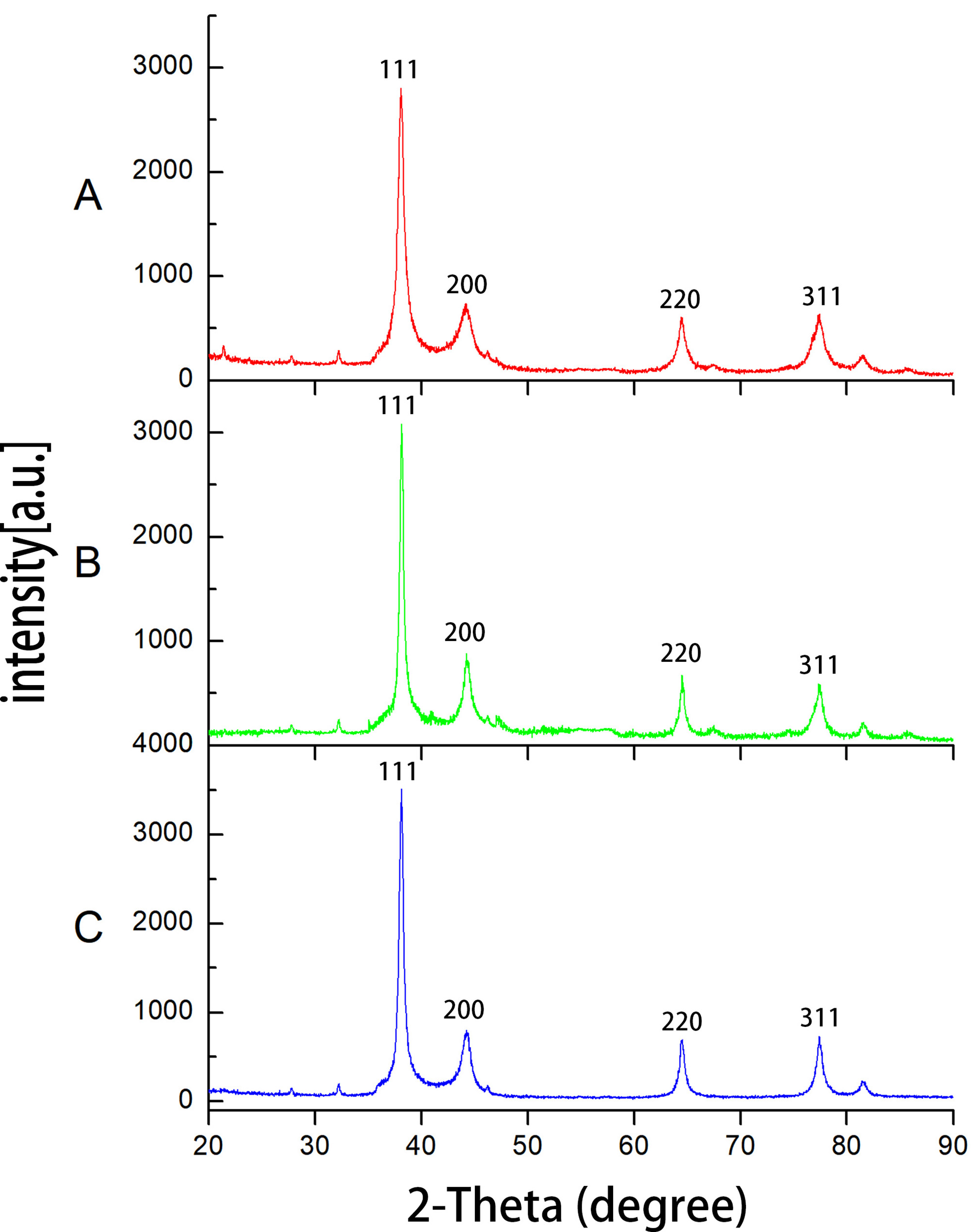

Supplement: Supplementary file 1 [file Data_Sheet_1.zip › supplymentary materials/supplementary Figure/Figure S3.jpg]

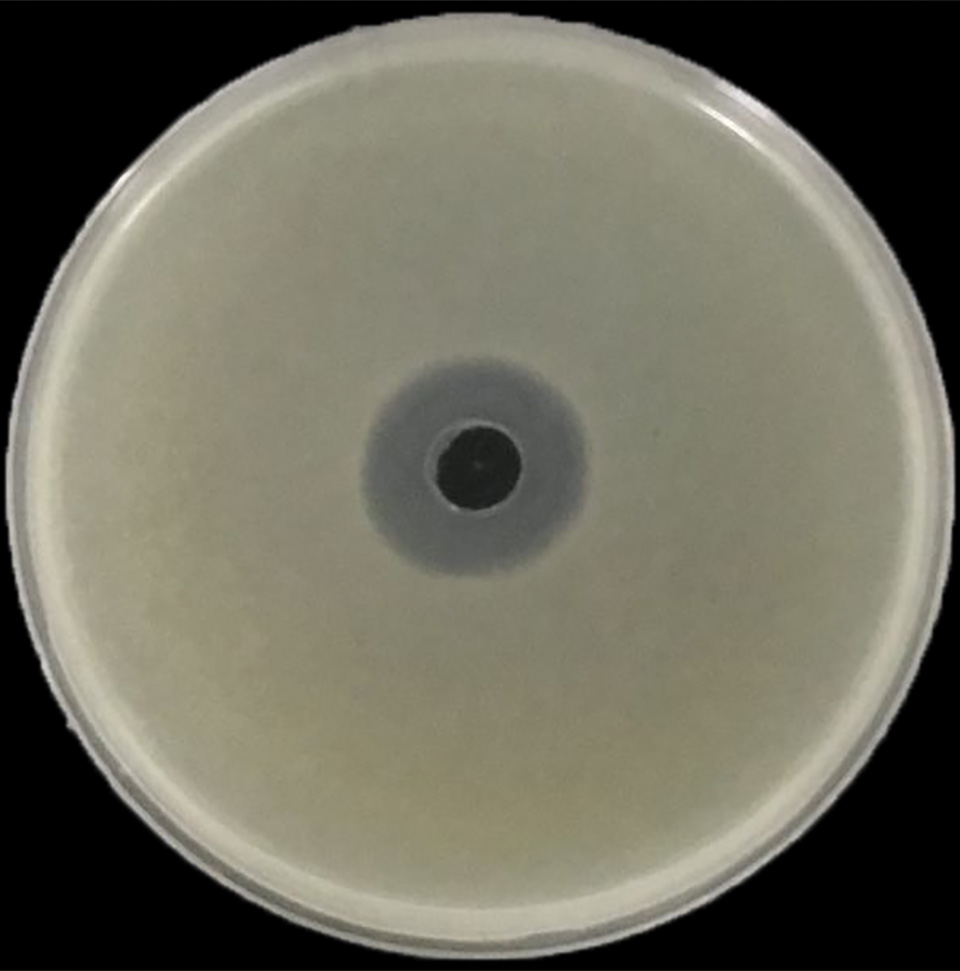

Supplement: Supplementary file 1 [file Data_Sheet_1.zip › supplymentary materials/supplementary Figure/Figure S4.jpg]

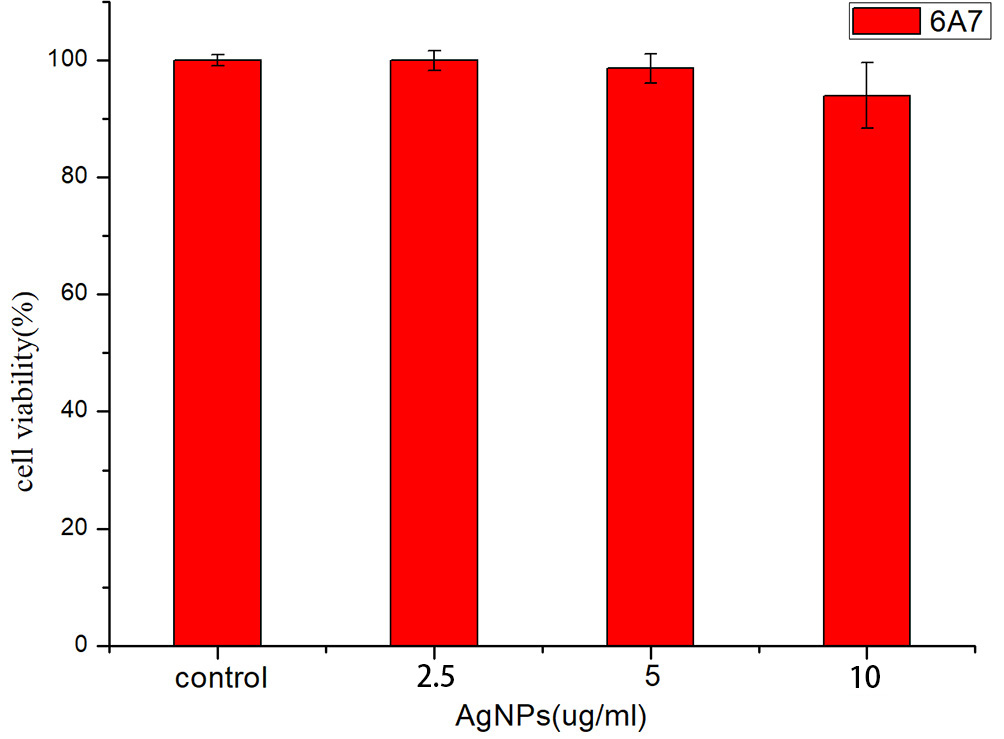

Supplement: Supplementary file 1 [file Data_Sheet_1.zip › supplymentary materials/supplementary Figure/Figure S5.jpg]
