# Supplementary material for: Phytofabrication of Silver Nanoparticles Using Three Flower Extracts and Their Antibacterial Activities Against Pathogen Ralstonia solanacearum Strain YY06 of Bacterial Wilt
Source: Front Microbiol. 2020 Sep 15;11:2110. doi: 10.3389/fmicb.2020.02110 (PMC7522305; doi:10.3389/fmicb.2020.02110)
Supplement: Supplementary file 1 [file Data_Sheet_1.zip › supplymentary materials/supplymentary material.doc]

**Figure S1.** UV–Vis spectra of AgNPs synthesized by (A) Canna indica L. flower; (B) Cosmos bipinnata Cav. flower; (C) Lantana camara L. flower.

**Figure S2.** Fourier transform infrared spectra of AgNPs synthesized by (A) Canna indica L. flower; (B) Cosmos bipinnata Cav. flower; (C) Lantana camara L. flower.

Figure S3. XRD spectra of silver nanoparticles synthesized by (A) Canna indica L. flower; (B) Cosmos bipinnata Cav. flower; (C) Lantana camara L. flower.

**Figure S4.** In vitro inhibitory effect of silver nanoparticles synthesized by flower extract against Xanthomonas oryzae pv. oryzae (Xoo) strain GZ 0003.

**Cell lines and cell culture media**

The Mouse hybridoma cells (6A7) were provided from the Institute of Biotechnology, Zhejiang University, Hangzhou, China. The cell were grown in medium RPMI - 1640.

**Cytotoxicity evaluation**

The Mouse hybridoma cells (6A7) were inoculated into 96-well plates with 5 × 10^3^ per well，and three different concentrations of silver nanoparticles mediated by *Lantana camara L.* flower were added to each well after cells incubated overnight. After The cells sample were incubated for 24h at 37℃ under atmosphere (5% CO_2_), The culture medium was removed and 100ul of the fresh medium with 10% CCK-8 were inoculated into wells for 4 h at 37℃. Absorbance was measured at a wavelength of 450 nm by using microplate reader. The cell viability was evaluated as a percentage of OD assay/OD control. There were three replicates for each treatment and three repeats for each experiment.

**Figure S5.** Cell viability assay in mammalian cells incubated with AgNPs. 6A7 cells were treated with different concentrations of silver nanoparticles for 24 h.
